# Patterns of Population Genomic Variation and Evolutionary History of European Hake in the Northeastern Atlantic

**DOI:** 10.1002/ece3.73085

**Published:** 2026-02-13

**Authors:** José Martin Pujolar, Courtney E. C. Gardiner, Sophie von der Heyden, Joana I. Robalo, Rita Castilho, Regina L. Cunha, Dorte Meldrup, Romina Henriques, Einar E. Nielsen

**Affiliations:** ^1^ Centre for Gelatinous Plankton Ecology and Evolution DTU Aqua – Technical University of Denmark Kongens Lyngby Denmark; ^2^ Department of Botany and Zoology Stellenbosch University Matieland South Africa; ^3^ MARE – Marine and Environmental Sciences Centre, ARNET‐Aquatic Research Network ISPA Instituto Universitario Lisbon Portugal; ^4^ Universidade do Algarve – Campus de Gambelas Faro Portugal; ^5^ Centre of Marine Sciences (CCMAR), Campus de Gambelas Faro Portugal; ^6^ Pattern Institute Faro Portugal; ^7^ Section for Marine Living Resources DTU Aqua – Technical University of Denmark Silkeborg Denmark; ^8^ Department of Biochemistry, Genetics and Microbiology, Faculty of Natural and Agricultural Sciences University of Pretoria Hatfield South Africa

**Keywords:** climate change, diversity, marine ecosystems, population genomics, range expansion

## Abstract

Climate change is driving species to shift their distribution ranges, potentially altering the level of genomic structuring and connectivity between populations. Additionally, fishing practices might further reduce genomic diversity and limit the potential adaptability of species to environmental changes. We use whole‐genome sequencing for the first time to explore current and historical patterns of genomic diversity in European hake ( *Merluccius merluccius* ) from the Northeast Atlantic, focusing on the recently expanded distribution range in the North Sea. Genomic data revealed a complex scenario in the North Sea and neighbouring regions, with three distinct populations: North Sea, Celtic Sea and Portugal. Individuals from the Kattegat, Skagerrak and west coast of Denmark were highly differentiated from those in the Celtic Sea and waters around Ireland. The Northern North Sea appears as a transition zone, with individuals from higher latitudes assigned to the Celtic Sea group and those from lower latitudes to the North Sea group. The more distant Portuguese individuals appeared as a third distinct population. Although the differentiation among these populations was shallow when the entire dataset was used, a subset of 99,364 outlier markers revealed a much deeper divergence. Demographic analyses indicated that these populations are relatively young and have large effective population sizes and thus without sufficient time to build a signature of differentiation by genetic drift. At the same time, selection for local adaptation is strong enough to overcome the effects of contemporary gene flow. Our findings have important implications for managing the European hake stocks in the Northeastern Atlantic, highlighting the need for management measures that address shifts in species and population distribution due to climate change, as well as needing to account for different populations contributing to fisheries within a single stock. Preserving the genomic diversity within and among fish stocks is crucial for maintaining the long‐term resilience of marine ecosystems and the services they provide.

## Graphical abstract

We use for the first time whole‐genome sequencing to explore current and historical patterns of genetic diversity in European hake in the Northeastern Atlantic, focusing on the recently expanded distribution range in the North Sea. Genomic data revealed a complex scenario with three populations: North Sea, Celtic Sea and Portugal. Demographic analyses point to young populations, with shallow differentiation when using all genomic markers but a deeper divergence when using outlier markers.

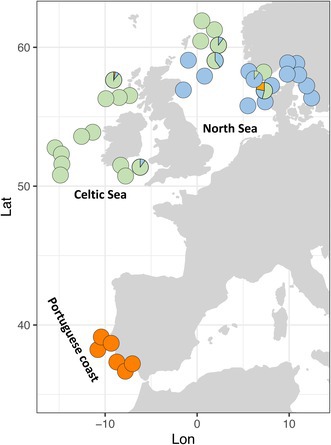

## 1 Introduction

The interplay between geographic connectivity and isolation plays a critical role in shaping the evolutionary dynamics of marine species (Hemmer‐Hansen et al. 2007; Potkamp and Fransen 2019; Faria et al. 2021). Populations of many marine organisms are demographically and genetically interconnected, resulting from either passive dispersal of planktonic larval stages or active migration of juveniles and adults (Nielsen et al. 2005; Bradbury et al. 2008; Cowen and Sponaugle 2009; Chust et al. 2016). The degree of separation between populations can vary significantly depending on population size and the dispersal capacity of species, with high gene flow expected to prevent populations from following independent evolutionary trajectories (Riginos et al. 2014). On the contrary, barriers to connectivity and gene flow can drive the accumulation of genetic differences between populations over time and eventually result in genetically distinct populations (Hemmer‐Hansen et al. 2007). Barriers to gene flow in the marine environment include ocean currents, coastal fronts and upwelling systems, as well as environmental factors such as sea surface temperature and salinity (Cowen and Sponaugle 2009; Henriques et al. 2012; Nielsen et al. 2012; Burridge et al. 2015; Van der Ven et al. 2020; Choo et al. 2021). In addition, life‐history traits such as philopatry and natal homing to different spawning grounds can lead to isolated gene pools (Díaz‐Arce et al. 2023). Besides contemporary barriers to gene flow, historical climatic events, especially the advances and retreats of ice sheets during the last glacial period (20,000–100,000 years ago), have resulted in significant demographic changes, range expansions, the emergence of newly genetically isolated populations and even speciation events (Hewitt 1996, 2004; Alheit et al. 2012; Do Prado et al. 2018).

The evaluation of connectivity and gene flow is particularly relevant in the context of climate change and shifting marine fish populations that might be commercially exploited, which will impact their management and utilisation. Anthropogenic climate change is driving species to shift their distributions, with temperate species often moving poleward as they track their thermal niches and expand into newly suitable habitats (Wisz et al. 2015; Pinsky et al. 2020). Moreover, overfishing is contributing to making exploited species more vulnerable to the effects of global climate change (Pinsky and Byler 2015). In combination, climate change and fishing pressure can impact the distribution of commercially exploited fish as illustrated in the case of North Sea cod ( *Gadus morhua* ), with the northward range shift in the last 100 years likely caused by rising sea temperatures and the eastward shift attributable to overfishing in the western and central North Sea (Engelhard et al. 2014; Heath et al. 2014). While global climate change drives species to new regions as they respond to changing environmental conditions, fishing impacts their resilience and abundance resulting in significant changes in fish community (Lees et al. 2006). This has been documented in the Northwest Atlantic (Frank et al. 2005), the North Pacific Ocean (Hsieh et al. 2005), the North Sea (Kirby et al. 2009), the Barents Sea (Aschan et al. 2014) and the Mediterranean Sea (Ouled‐Cheikh et al. 2022), among other regions.

The European hake ( *Merluccius merluccius* ) is a benthopelagic marine fish, widely distributed in the Northeast Atlantic Ocean, ranging from the coasts of Norway and Iceland to the Gulf of Guinea (Murua 2010). Historically, European hake populations were mainly concentrated in the Mediterranean and the eastern Atlantic, with their distribution extending as far north as the North Sea, Skagerrak and Kattegat (Casey and Pereiro 1995). In recent years, the European hake has increased in abundance and extended distribution into new regions of the North Sea, moving further north into the Norwegian Sea and eastward into the Baltic Sea (Baudron and Fernandes 2015; Cormon et al. 2014), being occasionally recorded in the Bornholm area (Heessen et al. 2015) and documented as far east as Lithuania (Bacevicius and Kregzdys 2017). Potential drivers for this range expansion include climate change, with increasing temperatures allowing hake populations to occupy new areas, as well as changes in ecosystem dynamics such as prey availability (Baudron and Fernandes 2015). From an ecological perspective, European hake plays a fundamental role in the marine trophic web as a high‐level top predator feeding on mesopelagic communities, such as anchovies ( *Engraulis encrasicolus* ) and sardines ( *Sardina pilchardus* ), regulating their abundances and maintaining ecological balance and biodiversity (Coll et al. 2016). European hake is one of the most commercially important marine species in the Northeast Atlantic and Mediterranean fisheries and has been intensively exploited for centuries (Izquierdo et al. 2021; Morales‐Nin et al. 2022). The management of the European hake in the North Atlantic region has been the subject of ongoing discussion, particularly regarding the International Council for the Conservation of the Seas (ICES) classification of two distinct fishing stocks: a Northern stock that encompasses the Norwegian Sea, the North Sea, Skagerrak and Kattegat, the Celtic Sea and the Bay of Biscay; and a Southern stock that includes the Cantabrian Sea and the Iberian West Coast, extending south to Gibraltar. The two stocks are delineated at Cap Breton, located between the southern Bay of Biscay and the eastern Cantabrian Sea, which has traditionally been considered a natural barrier that may limit the exchange of individuals between regions (ICES 2022a, b). However, genetic data do not provide evidence to support the boundary between the Northern and Southern stocks at Cap Breton, as previous genetic studies revealed no differences among Atlantic Iberia‐Biscay‐Irish Sea populations, but a more complex population structure within the Northeastern Atlantic than the two discrete stocks proposed by ICES (Roldán et al. 1998; Pita et al. 2011, 2014, 2016; Milano et al. 2014; Westgaard et al. 2017; Leone et al. 2019).

However, previous work relied on either a small number of molecular markers (such as mitochondrial DNA and nuclear microsatellite loci) or reduced‐representation sequencing. These approaches might not contain enough statistical power to detect subtle levels of local adaptation or population divergence. Therefore, the aim of our study is to use, for the first time, whole‐genome sequencing to expand the current knowledge on the population structure of European hake in the northeastern Atlantic, notably adding valuable data from its northern range (North Sea and neighbouring areas) to support ongoing efforts in this region to delineate population structure and fisheries management. The detailed analyses of areas connected to the North Sea are particularly relevant given the recent increase in biomass and the unclear origin of these individuals. We analysed millions of genome‐wide single nucleotide polymorphisms (SNPs) to investigate patterns of genomic variation within and across regions and to assess population structure and gene flow. Connectivity at the northern edge of the distribution is critical yet not evaluated, especially in a context of climate change and shifting populations, which has important implications for fishery dynamics and stock management. Therefore, we integrate historical and contemporary demographic processes to better understand population connectivity over time. Using a coalescent approach, we reconstruct the past demographic history of the species in the Northeastern Atlantic, with particular focus on the northern expanded range of the species. We also identify genomic regions showing elevated differentiation across the genome and explore the functional annotations of genes located within those regions, which might reflect adaptation to local environmental conditions.

## 2 Materials and Methods

### 2.1 Sampling

**FIGURE 1:**
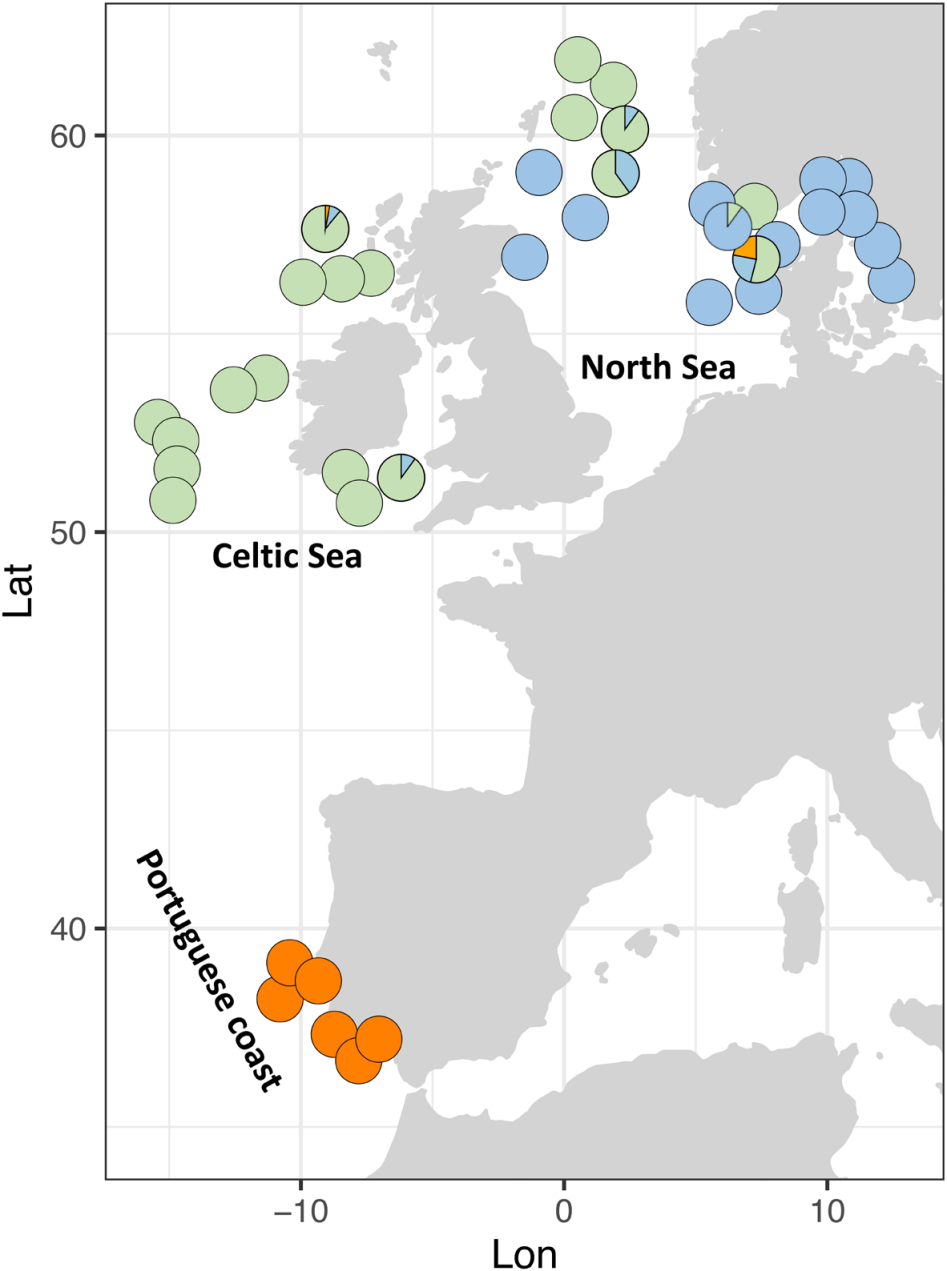
Sampling locations of 40 individuals of European hake collected from Denmark, the Northern North Sea, Ireland and Portugal. Each circle represents one individual, with colours showing the proportion of ancestry assigned to each cluster (North Sea, blue; Celtic Sea, green; Portugal, orange) as inferred by admixture analysis using 99,364 outlier SNPs. Individuals with mixed ancestry are represented by circles with multiple colours corresponding to their relative contributions from each cluster. See Table S1 for sampling details.

A total of 40 European hake *Merluccius merluccius* individuals were collected during 2021 from four regions: Denmark, Northern North Sea, Ireland and Portugal (Table S1; Figure 1) as part of national fish monitoring survey programs. Samples from Denmark (*N* = 13) were collected from the Kattegat, the Skagerrak and the west coast of Denmark. Northern North Sea samples (*N* = 8) were collected off the east coast of Scotland and in Norwegian waters of the northern central North Sea. Samples from Ireland (*N* = 13) were collected along the northern coast, the Porcupine Bank west of Ireland and the southern coast in the Celtic Sea. Samples from Portugal (*N* = 6) were collected from the Lisbon Coast in western Portugal, as well as from Faro and the Gulf of Cadis in southern Portugal.

### 2.2 Sequencing

Muscle tissue was collected onboard from each individual and stored in a ‒20°C freezer until DNA extraction. Genomic DNA was extracted using the EZNA Tissue DNA Kit, with subsequent quality control via electrophoresis on 1% agarose gel and measurement of the DNA concentration using a Qubit 3.0 (Qubit dsDNA BR Assay kit). DNA extracts were fragmented and used to construct short‐insert libraries of circa 350 base‐pairs (bp). A standard library‐construction protocol was used that included end‐repair, A‐tailing, adapter ligation, PCR amplification and size selection. The resulting libraries were then sequenced as 150 bp paired‐end reads for all 40 individuals on a DNBseq (DNA Nanoball Sequencing) platform at BGI Genomics, China.

### 2.3 Data Filtering and SNP Discovery

Data quality of the reads was initially inspected using FastQC (http://www.bioinformatics.babraham.ac.uk/projects/fastqc) and the FASTX‐Toolkit (http://hannonlab.cshl.edu/fastx‐toolkit). Next, adapters and low‐quality bases were removed with Trimmomatic v0.36 (Bolger et al. 2014) using a minimum Phred score of 15. Subsequently, paired and unpaired reads were aligned separately using BWA v0.7.1 (Li and Durbin 2009) to the chromosome‐level genome assembly of *M. merluccius* fMerMel1.1 (Martínez Portela et al. 2024; accession number GCA_964660975.1). The aligned data were pre‐processed using the Picard v2.6.0 toolkit (http://broadinstitute.github.io/picard), which involved merging, coordinate‐sorting and removing duplicate reads that may have resulted from PCR amplification during library preparation. A variant calling file (vcf) file with all SNP variants for all 40 individuals was constructed using the mpileup and call commands in BCFtools v1.9 (Li et al. 2009). Subsequently, SNP variants were filtered using vcftools v0.1.14 (Danecek et al. 2011), retaining only biallelic SNPs present in all 40 individuals sequenced (i.e., zero missing data), with a minimum quality score of 20, a total read depth between 400 and 4000 (summed across all 40 individuals) and with a minor allele frequency (MAF) greater than 0.05 to avoid potential bias from rare alleles (Hemstrom et al. 2024).

As a final step, we produced a filtered SNP dataset after linkage disequilibrium‐based (LD) variant pruning implemented in PLINK v1.9 (Purcell et al. 2007). The indep‐pairwise option was used with a window size of 100, a variant count of 5 to switch window at the end of each step and a *r* ^2^ threshold of 0.5. The pruned LD dataset was used for downstream analyses sensitive to LD such as PCA, Structure, *F*_ST_ and nucleotide diversity calculations, while the unpruned LD dataset was used for the genome scans.

### 2.4 Detection of Outlier SNPs

Outlier detection at the whole‐genome level was performed using pcadapt v.4.4.0 (Privé et al. 2020) to identify potentially non‐neutral markers. An advantage of pcadapt is that no predefined populations are needed and the detection does not rely on *F*_ST_ values. The optimal number of principal components (K) was determined using a scree plot with up to 10 Ks and choosing K on the basis of the variance drop‐off. Outlier SNPs were identified based on *p*‐values adjusted using the Benjamini‐Hochberg correction to account for multiple testing (*p* < 0.1).

### 2.5 Genome‐Wide Diversity and Population Structure Analyses

All analyses were performed on three different SNP datasets: the full dataset, the subset of outlier SNPs and the subset of neutral SNPs (i.e., not identified as outliers). Levels of genomic diversity were assessed using Nei and Li (1979) nucleotide diversity (π) and individual observed heterozygosity (H_o_) calculated using vcftools v0.1.14 (Danecek et al. 2011). Standard errors and confidence intervals were obtained by performing a bootstrap function with 1000 replicates using the boot package in R. Diversity values across populations were compared by one‐way ANOVA using R. Standardised genomic differentiation (*F*_ST_) statistics between population pairs were calculated using vcftools v0.1.14 following Weir and Cockerham (1984). Significance of pairwise *F*_ST_ values was assessed by bootstrapping over loci to generate a confidence interval around the observed *F*_ST_. *p*‐values were calculated using a one‐sample *t*‐test for each pairwise *F*_ST_.

Population structure was initially explored with a principal component analysis (PCA) using the program smartPCA from the Eigensoft package (Patterson et al. 2006). Population structure was further investigated using ADMIXTURE (Alexander and Lange 2011), which uses a clustering algorithm to infer the most likely number of groups (K) and then calculates the proportion of ancestry components (admixture proportion) for each individual. Following the software guidelines, the cross‐validation (CV) procedure was applied to choose the best K for the model. The analysis was performed with *K* = 1–6, with 10 independent runs for each K to check the consistency of results.

### 2.6 Genome Scans and Functional Analysis of Candidate Genes

Genome scans were conducted to detect ‘genomic islands of differentiation’, as defined by peaks in pairwise *F*_ST_ (Wolf and Ellegren 2017). The method is based on the use of summary statistics in windows across the genome, with regions with very high *F*_ST_ relative to the background assumed to be under strong local adaptation (Beaumont and Balding 2004). After testing different window sizes that rendered similar results, we calculated a sliding‐window *F*_ST_ with a window size of 10 kb using VCFtools v0.1.14 (Danecek et al. 2011) with the unpruned LD dataset. Only windows with more than 10 SNPs were considered to obtain reliable *F*_ST_ estimates. Windows with average window *F*_ST_ values above the 99th percentile of the empirical distribution were retained and considered as candidate regions putatively under selection. Sliding‐window *F*_ST_ along chromosomes was visualised with a Manhattan plot using CMPlot in R.

Gene predictions for the *M. merluccius* reference genome (Martínez Portela et al. 2024) were used to establish the genomic position of the candidate SNPs for local selection. Gene descriptions were obtained from the Zebrafish Information Network (ZFIN) database (http://zfin.org), together with human orthologs, which were used in the GeneCards human gene database (http://genecards.org) to retrieve the Entrez gene summary, the UniProtKB/Swiss‐Prot summary as well as the Ensembl Gene IDs. Functional interpretation of the set of candidate genes was obtained using the Gene Ontology (GO) term classification and Kyoto Encyclopedia of Genes and Genomes (KEGG) pathway enrichment analysis implemented in DAVID v6.8 (Dennis et al. 2003). Standard settings (minimum gene count = 2 and EASE score = 0.1) in DAVID were used, and *p*‐values were adjusted for multiple testing using the Benjamini‐Hochberg method. Enriched GO terms were further analysed with the ClueGo plug‐in of Cytoscape v3.8.2 (Bindea et al. 2009), which visualises the non‐redundant biological terms for large clusters of genes in a functionally grouped network. Analyses were conducted using the Ensembl gene IDs, only considering GO terms with corrected *p*‐values.

Lastly, we tested whether genes known to be involved in osmoregulation (Stern and Lee 2020) were located in regions putatively under selection characterised by high values of genomic differentiation. Osmoregulation plays a critical role in maintaining osmotic balance in marine organisms, particularly in environments with salinity gradients (Lee 2023). Given the ecological differences between the North Sea and neighbouring North Atlantic waters, including salinity and temperature, we hypothesised that divergence in osmoregulatory genes may contribute to local adaptation. Focusing on these genes allows us to test whether salinity is a potential driver of genomic differentiation in this system. The following genes were tested: sodium/potassium‐transporting ATPase subunit alpha (ATP1A1a) and beta (ATP1A1b), Na(+)/H(+) exchange co‐factor regulatory (NHE), V‐type proton ATPase, carbonic anhydrase and acid‐sensing (proton‐gated) ion channel.

### 2.7 Demographic History Inference

Coalescent methods can be used to infer past population dynamics and demographic changes over historical time scales with high resolution. In our study, we applied the Pairwise Sequentially Markovian Coalescent (PSMC) method (Li and Durbin 2011) to infer the demographic history of the European hake. PSMC extracts demographic information from the distribution of heterozygous sites across the genome and estimates the distribution of the time since the most recent common ancestor (TMRCA) for each allele pair at all loci in the genome of a single individual. This approach provides insights into how effective population sizes (N_E_) have changed over time.

For all individuals, a consensus sequence was generated with BCFtools v1.9, following the same approach as Nadachowska‐Brzyska et al. (2015). First, variants were called for each individual separately with the mpileup and call commands. Subsequently, the consensus command was used to create a consensus sequence. The resulting vcf was converted to the psmc input format with vcfutils.pl. (distributed with BCFtools). Next, the consensus sequence was divided into non‐overlapping 10 bp bins, with bins scored as either homozygous or heterozygous. Then, PSMC v0.6.5 (Li and Durbin 2011) was run with 25 iterations, Tmax (‐t) of 15, initial mutation/recombination ratio (‐r) of 5 and time bin parameter (‐p) set to the standard ‘4 + 25*2 + 4 + 6’. For parameter conversion, results were scaled using a conservative average mutation rate of 1 × 10^−8^ per nucleotide per generation (Nikolic et al. 2020). For PSMC scaling, we assumed a generation time of 3.2 years, corresponding to the age at 50% sexual maturity for European hake (Pinerio and Sainza 2003). While mean reproductive age may be higher in this iteroparous species, age at maturity is the only well‐established reproductive timing metric available in the primary literature for European hake. Moreover, alternative assumptions would proportionally rescale the timing but not the inferred demographic trends.

## 3 Results

### 3.1 SNP Datasets

Whole‐genome re‐sequencing of 40 European hake individuals generated on average 72.7 million reads of 90 bp per individual, after adapter removal and quality trimming. Filtering of low‐quality reads led to the exclusion of an average of 8.9% of reads (Table 1). Retained sequences (91.1%) had a mean depth of 24.6× (19.1–30.5×) and a mean quality score of 36.4. On average, 94.9% of paired sequences and 91.2% of unpaired sequences aligned to the *M. merluccius* genome assembly, with a mean mapping quality of 40.2. After mapping, a total of 23,125,767 SNPs were obtained. After filtering for minimum depth, quality and biallelic SNPs genotyped in all 40 individuals, a total of 8,524,274 SNPs was obtained. After filtering for linkage disequilibrium, a full dataset of 5,001,966 high‐quality SNPs was retained for all downstream analyses except the selection tests, which required the unpruned dataset. Using pcadapt with *K* = 2, a total of 99,364 outlier SNPs were identified based on *p*‐values adjusted using the Benjamini‐Hochberg correction and constituted the outlier‐only dataset. Outlier SNPs were distributed all across the genome.

**TABLE 1:** Statistics describing the distribution of different properties of whole‐genome sequences for 40 individuals (averaged) after each filtering and alignment step to the European hake genome assembly using Trimmomatic, Bowtie and Samtools.

Trimmomatic	N reads	%
N initial reads	72,659,760	
Both surviving	52,936,186	72.74
Forward only surviving	7,384,340	10.21
Reverse only surviving	5,825,737	8.04
Retained	66,146,264	91.11
Dropped	6,445,740	8.89

BWA	N reads	%
N paired reads	106,592,583	
N mapped	101,103,065	94.85
N unpaired reads	12,884,966	
N mapped	11,751,088	91.2

Samtools	Mean	SD
Coverage	94.43	0.8
Mean depth	24.59	3.38
Mean base Q	36.41	0.84
Mean map Q	40.15	1.15

Abbreviation: SD, standard deviation.

### 3.2 Genome‐Wide Diversity and Patterns of Population Structure

Analyses of genomic diversity revealed similar diversity levels among the northern locations (Denmark, Northern North Sea and Ireland), with comparable values for both nucleotide diversity (π = 0.255–0.259) and observed heterozygosity (*H*_o_ = 0.162–0.169) (Table S2). In comparison, Portugal exhibited a significantly higher heterozygosity (*H*_o_ = 0.177; *p* = 0.002) and a higher nucleotide diversity (π = 0.263), although the latter was not statistically significant.

**FIGURE 2:**
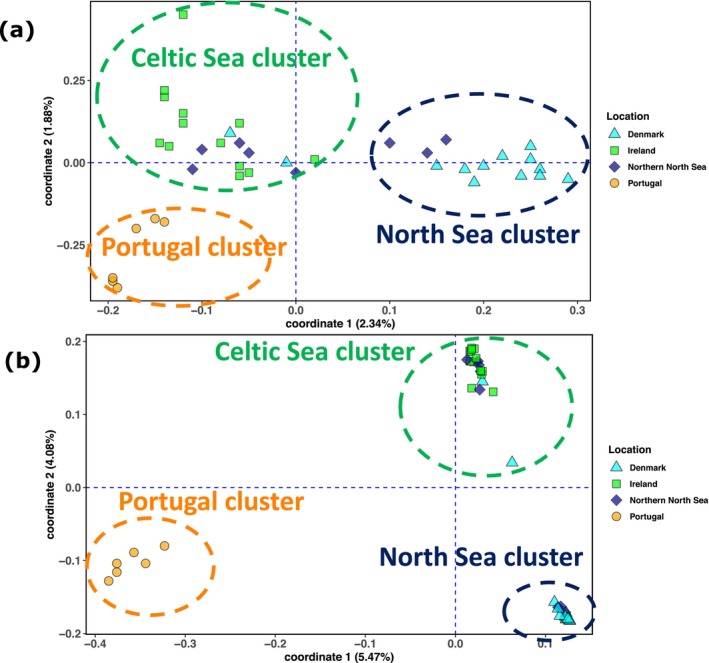
Visualisation of population structure using a principal component analysis (PCA) of all 40 individuals using (a) all SNPs and (b) a subset of 99,364 outlier SNPs. Each point represents an individual. Dashed circles are manually drawn to indicate the groups identified for visual guidance only.

The analysis of PCA on the full dataset identified three distinct clusters: (i) a ‘North Sea’ cluster including individuals from around Denmark (Kattegat, Skagerrak and west coast of Denmark) and some of the northern North Sea individuals, (ii) a ‘Celtic Sea’ cluster including all individuals from the northern, western (Porcupine Bank) and southern coasts of Ireland and some of the northern North Sea individuals and (iii) a ‘Portugal’ cluster consisting exclusively of individuals from Portugal (Figure 2a). Some mixing of individuals was apparent, as two individuals sampled off the west coast of Denmark grouped within the Celtic Sea cluster. Samples from the northern North Sea split into the two clusters according to latitude, with the northernmost individuals clustering within the Celtic Sea cluster, while the three southernmost individuals clustered within the North Sea cluster. The PCA based on the subset of 99,364 outlier SNPs revealed the same three well differentiated clusters with greater resolution (Figure 2b). The subset of neutral SNPs (excluding outliers) showed no clear clustering among groups (Figure S1). Pairwise *F*_ST_ values among the three differentiated clusters were significant, ranging from 0.005 to 0.007 and from 0.133 to 0.191 (Table 2) when using all loci and only the subset of outlier SNPs, respectively.

**TABLE 2:** Pairwise genomic differentiation (*F*_ST_) considering all SNPs (below diagonal) and considering only the subset of 99,614 outlier SNPs (above diagonal) between (a) all sampling locations and (b) the North Sea, Celtic Sea and Portugal clusters. Significant values in bold.

*F*_ST_	Denmark	N North Sea	Ireland	Portugal
Denmark	*****	**0.025**	**0.110**	**0.165**
N North Sea	0.002	*****	**0.028**	**0.126**
Ireland	0.003	0.002	*****	**0.154**
Portugal	**0.006**	**0.005**	**0.005**	*****

*F*_ST_	North Sea cluster	Celtic Sea cluster	Portugal cluster
North Sea cluster	*****	**0.133**	**0.191**
Celtic Sea cluster	**0.005**	*****	**0.154**
Portugal cluster	**0.007**	**0.005**	*****

**FIGURE 3:**
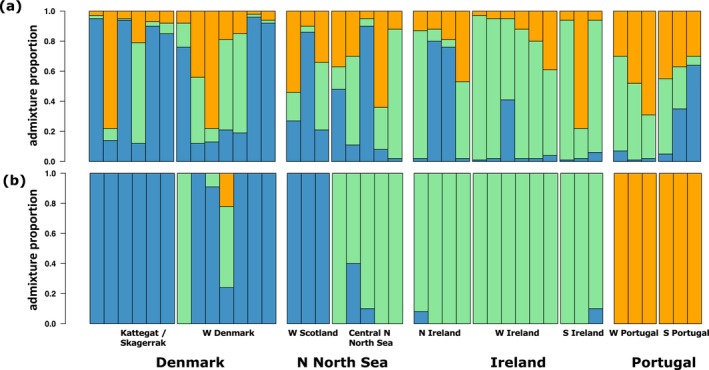
Graphical representation of the Bayesian clustering approach implemented in ADMIXTURE based on (a) all SNPs and (b) a subset of 99,614 outlier SNPs. Most likely scenario is three clusters (*K* = 3) for both datasets, based on the cross‐validation (CV) procedure. Individual admixture proportions are shown for each individual.

Admixture analyses were performed on LD‐pruned datasets filtered for MAF > 0.05. We tested three SNP datasets: all SNPs, neutral SNPs and outlier SNPs. Using the best *K* value (*K* = 3; Table S3), analyses based on the subset of outlier SNPs revealed three well differentiated clusters, congruent with the PCA results (Figure 3). Among the samples from Denmark, all individuals from Kattegat/Skagerrak were non‐admixed. One individual from the West coast was 100% assigned to the Celtic Sea cluster, while another was highly admixed. In the northern North Sea, individuals from lower latitudes were assigned to the North Sea cluster, while those from higher latitudes were assigned to the Celtic Sea cluster. Among the samples from Ireland, individuals from Porcupine Bank were all non‐admixed, while some admixed individuals were observed along both the northern and southern coasts. No admixture was observed among samples from Portugal. Admixture analyses based on neutral and all SNPs (Figure 3; Table S3) produced broadly admixed ancestry profiles.

### 3.3 Genome‐Wide Selection and Candidate Gene Analyses

**FIGURE 4:**
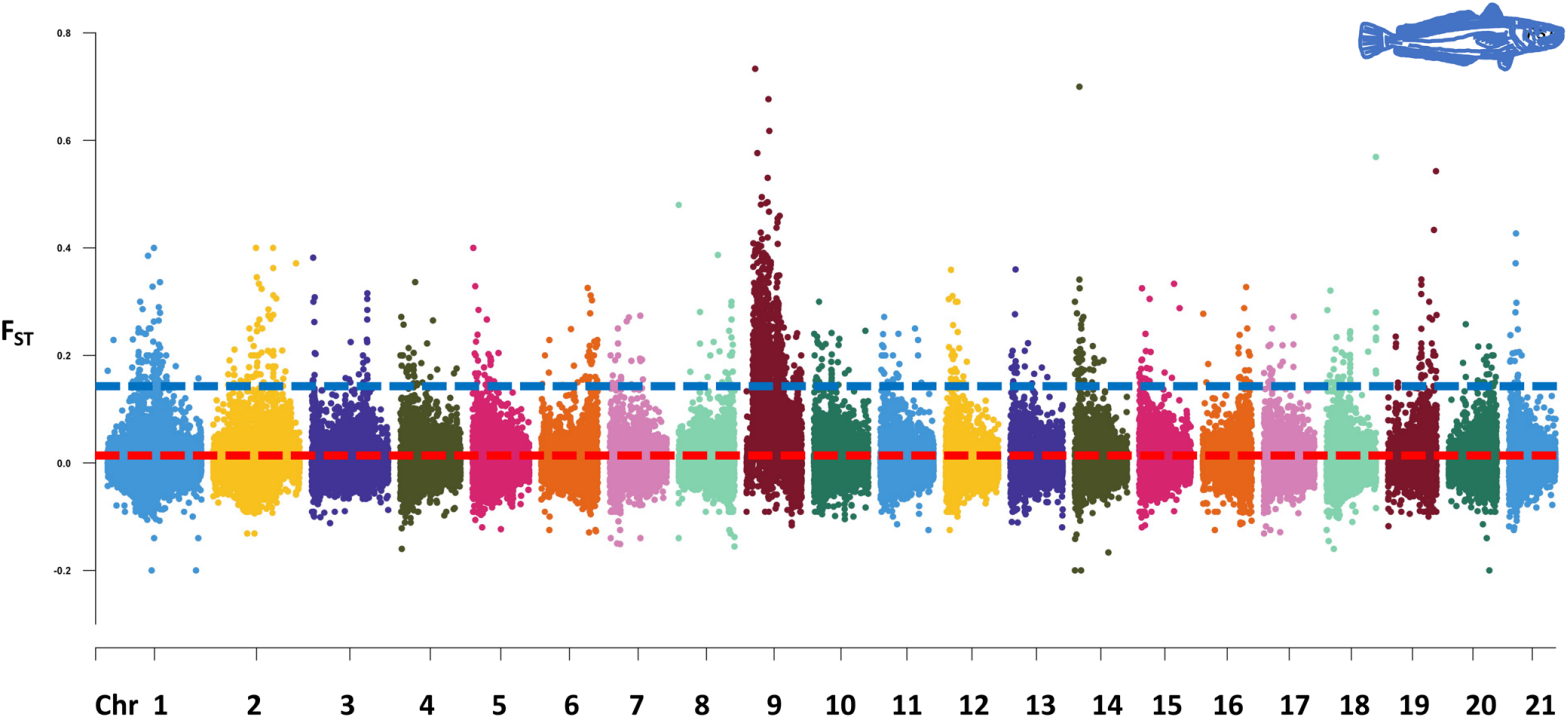
Manhattan plot based on sliding‐window genetic differentiation FST comparing the North Sea and Celtic Sea clusters. The average FST is represented by a discontinuous red line. Regions above the 99% percentile of the empirical distribution are marked with a discontinuous blue line.

Genome scans were used to identify ‘islands of differentiation’ across the entire genome when comparing the North Sea and Celtic Sea clusters, as the focus of the study was to compare the North Sea with neighbouring regions. While windows with *F*_ST_ values above the 99th percentile of the empirical distribution were observed across all chromosomes, chromosome 9 stood out, exhibiting an unexpectedly high number of regions with elevated *F*_ST_ (Figure 4). These regions on chromosome 9 accounted for 56.9% of all regions putatively under selection, including several regions spanning over 100 kb. By comparison, no other chromosome contributed more than 5% of the total regions under selection.

**FIGURE 5:**
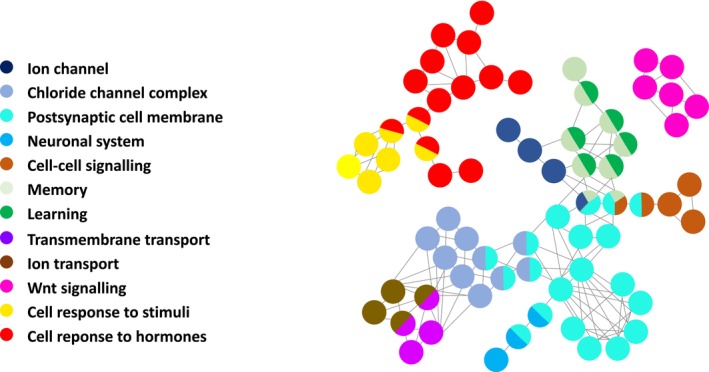
ClueGO network visualisation of functional terms identified in Gene Ontology (GO) and KEGG pathway analysis comparing the North Sea and Celtic Sea clusters. Analyses included biological processes, cellular components and metabolic and signalling pathways. Analyses were conducted only considering GO terms with corrected *p*‐values < 0.05 and selecting the most significant GO term as the representation of the group. Each circle represents a functional term, grouped by colour to indicate related functional terms or pathways. Lines connecting circles indicate shared genes between functional terms.

Regions identified under selection encompassed a total of 484 genes, which were used for enrichment analysis. The enriched pathways included chloride channel complex (associated with osmoregulation), postsynaptic cell membrane, neuronal system, memory, learning, transmembrane transport, Golgi, endoplasmic reticulum, thermogenesis, Wnt signalling and cell response to stimuli (Figure 5). Genes putatively under selection within the chlorine channel pathway included CFTR (CF Transmembrane Conductance Regulator) and CLCN2 (Chloride Voltage‐Gated Channel 2). A list of all annotated genes including summaries from Entrez and UniProtKB/Swiss‐Prot is provided in File S1. Beyond chloride channels, further evidence for selection in genes involved in osmoregulation included V‐type proton ATPase ATP6V1A, situated in a region with an *F*_ST_ value above the 99th percentile of the empirical distribution (*F*_ST_ = 0.22; Table S4). Other osmoregulatory genes such as the sodium/potassium pump ATP1A1b or carbonic anhydrase showed *F*_ST_ values > 0.5 but were not above the 99th percentile threshold.

### 3.4 Demographic Inference Using PSMC

**FIGURE 6:**
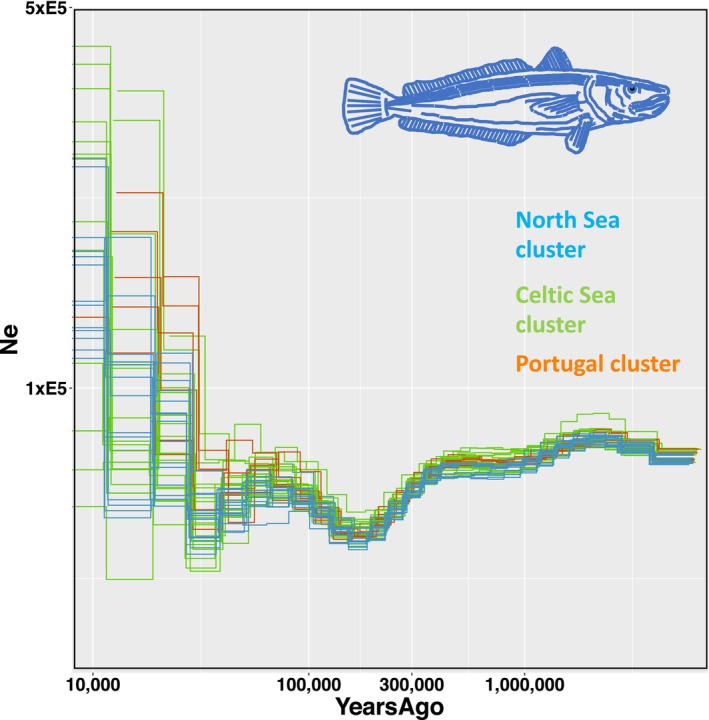
Demographic history reconstruction of European hake populations inferred using the PSMC (Pairwise Sequentially Markovian Coalescent) method. The three clusters are colour‐coded: North Sea (blue), Celtic Sea (green) and Portugal (orange). A universal mutation rate of 1 × 10^−8^ and an average generation time of 3.2 years were assumed.

The demographic history of European hake was reconstructed using Pairwise Sequentially Markovian Coalescent (PSMC). The inferred demographic history encompassed the time period from approximately 6 million years ago to 10,000 years ago (Figure 6). The initial effective population size was estimated at ca. 75,000 individuals, with a slight population expansion during the Pliocene (2.6–5.3 million years ago) followed by a prolonged period of demographic stability during the Pleistocene (beginning ca. 2.6 million years ago). The most significant demographic change occurred approximately 300,000 years ago in the middle‐late Pleistocene, marked by a population decrease to ca. 50,000 individuals. In the early late Pleistocene, ca. 130,000 years ago, the species experienced a rapid population increase, regaining its original size. From ca. 100,000 years ago, the population remained stable throughout the last glaciation (20,000–100,000 years ago). Population sizes have generally increased in the most recent 20,000 years, particularly Portugal. However, the variability observed among individuals within populations during this period suggests that recent demographic trends are uncertain and should be interpreted with caution. In contrast, the demographic history of the species further back in time appears to be robust, with good agreement between the PSMC plots derived from the 40 individuals from the study, showing consistent demographic trajectories. These plots only diverge in the most recent time period since ca. 20,000 years ago. The PSMC suggests that the three hake populations identified in our study are apparently very young and were a single population until recently.

## 4 Discussion

### 4.1 A Complex Scenario in the Northeastern Atlantic With Three Distinct Populations

Characterising the population structure of marine species is essential for assessing and predicting the impacts of global climate change and fisheries on ecosystem diversity (Andersson et al. 2024). This becomes even more relevant in ecosystems where species are shifting their ranges in response to environmental changes, such as temperature and salinity (Alheit et al. 2012; Wisz et al. 2015). Our study, using whole‐genome sequencing for the first time, expands the current knowledge on the population structure of European hake in the northern Atlantic, revealing a complex scenario in the North Sea and neighbouring regions, with three distinct groups: North Sea, Celtic Sea and Portugal (Figure 1). When using all genomic markers, we identified shallow genetic differentiation among groups, hereafter referred to as populations for simplicity, but it became much stronger when using the subset of 99,364 outlier markers. Individuals from the Kattegat, Skagerrak and west coast of Denmark were highly differentiated from those in the Celtic Sea and waters off Ireland. The Northern North Sea appears as a transition zone, with individuals from higher latitudes assigned to the Celtic Sea population and those from lower latitudes to the North Sea population. The more distant Portuguese samples appeared as a third distinct population. Our genome‐wide study supports previous findings of genetic differentiation in *M. merluccius* in the Northeastern Atlantic. Using a panel of 381 SNPs, Milano et al. (2014) found weak differentiation at 299 neutral SNPs, while 7 outlier SNPs separated North Sea (east of Scotland), Celtic Sea and Northern Portugal. Using a panel of 53 SNPs, Westgaard et al. (2017) detected significant differentiation between Bay of Biscay and North Sea samples, while no differences were found within the North Sea (Kattegat vs. central North Sea). Using RAD‐sequencing, Leone et al. (2019) found significant differences between Bay of Biscay and Norwegian Sea samples, but their study did not include samples from the North Sea. While confirming these earlier studies, our study expands the geographic coverage and provides whole‐genome resolution, revealing more comprehensive patterns of differentiation. In particular, our analyses show that these groupings are not fully discrete, as evidenced by mixing between populations in the PCA and the presence of introgressed individuals in the admixture analysis. In future studies, the specific and detailed patterns of population distribution can be investigated in more detail with more individuals and lower coverage using a subset of the outlier SNPs (Nielsen et al. 2012). This would include key areas not sampled in our study such as the Bay of Biscay and the Cantabrian Sea. Given the relatively small sample size from Portugal in our study compared with the other regions, additional sampling would help verify the observed differentiation.

The identification of three differentiated populations of European hake (North Sea, Celtic Sea and Portugal) can to a large degree be explained by the reproductive behaviour of the species, which spawns in multiple discrete locations across its distribution range in the Northeastern Atlantic (ICES 2022b). The North Sea population likely corresponds to hake spawning in the northern part of the Kattegat and in the southern North Sea off the coasts of Belgium and the Netherlands (Svensson 2010; ICES 2022b), primarily during late winter and early spring, peaking in February–March. The Celtic Sea population likely corresponds to hake spawning on Porcupine Bank and the Great Sole off the Irish coast (ICES 2022c), mainly from December to March, peaking in January–February. The Portugal population likely corresponds to hake spawning off the Algarve and in the Gulf of Cadis (ICES 2022c), where spawning also occurs during winter, peaking between January and February, but takes place in comparatively warmer waters (12°C–14°C) than those associated with the more northern populations. The existence of separate spawning grounds in these three regions, North Sea, Celtic Sea and Portugal, strongly corroborates the three distinct populations of European hake in the Northeastern Atlantic identified in our study.

The shallow level of population divergence for hake in our study is expected in a highly fecund and abundant species with a pelagic early life history. Compared with other fish species expanding into the North Sea, this pattern aligns with that of sardines (McKeown et al. 2024), which also exhibit modest population divergence. In contrast, anchovies show a much deeper divergence (Pujolar et al. 2025), likely a result of environmental retention mechanisms and limited larval dispersal driven by oceanography and spawning timing (Huret et al. 2010). Sprat ( *Clupea sprattus* ) also shows strong genetic divergence in the North Sea area, but in this case, the differentiation is not between North Sea–Celtic Sea but involves a highly differentiated population in the Baltic Sea (McKeown et al. 2020).

Studies on population dynamics have shown how European hake is able to follow long migratory routes. Hake can travel from the Celtic Sea to the North Sea, entering the North Sea over the north of Scotland and continuing southwards following the Norwegian trench until the Kattegat (Baudron and Fernandes 2015). This contemporary connectivity could explain the low levels of genomic differentiation found in our study, which indicates that the North Sea includes a mix of local spawners and incoming migrants from the Celtic Sea spawning grounds. This is congruent with the study of Westgaard et al. (2017), comparing two groups of individuals collected in the Kattegat: large spawning hake collected during summer and juveniles and smaller sized adults collected during the non‐spawning season. Here, significant genomic differences were detected, with the two groups suggested to comprise individuals of local origin and migrants, respectively. A similar pattern has been observed in other fish species, such as the sister species shallow‐water hake ( *M. capensis* ) in southern Africa, which shows three populations potentially linked with different spawning grounds and differential migration abilities (Forde et al. 2023). As well as deep‐water hake ( *M. paradoxus* ) in southern Africa, which shows population substructuring and differential evolutionary pathways between males and females in the southern African region (Gardiner et al. 2025). In our study area, North Sea cod shows a combination of resident and migratory populations in the North Sea–Skagerrak–Kattegat area (André et al. 2016; Hemmer‐Hansen et al. 2019). Herring ( *Clupea harengus* ) also exhibits both resident and migratory behaviours in the Kattegat, with local spawning individuals co‐existing with individuals that spawn in the southwestern Baltic and the North Sea (Bekkevold et al. 2023).

### 4.2 Salinity‐Driven Local Adaptation on Osmoregulatory Genes

Our study provides evidence for putative signals of local adaptation in genomic variants located within osmoregulatory genes and pathways. Salinity differences across the distribution range of European hake likely drive spatially varying selection at these genes. Salinities are high in open waters around Ireland (> 35 PSU), where hake spawn at Porcupine Bank off the western coast and the Great Sole off the southern coast (ICES 2022c). Salinities are more variable in the North Sea (ICES 2023), around 34–35 PSU in the northern part, and around 30 PSU in southern spawning areas (i.e., English Channel, Wadden Sea), European hake spawns also in the Kattegat, a transitional area between the high saline North Sea and the low saline Baltic Sea, where surface salinity ranges from 18–26 PSU and bottom salinity from 32–34 PSU (Pacariz et al. 2014). Salinity is a key environmental factor exerting selective pressure on aquatic organisms and likely influencing local adaptation processes (Stern and Lee 2020). Osmoregulatory genes are expected to be under strong selection pressure, as documented in the invasion of freshwater habitats by native saline populations of the copepod *Eurytemora affinis* (Stern and Lee 2020), where ion transporter genes are the physiological targets of natural selection. Notably, ‘islands of genomic differentiation’ were detected predominantly on chromosome 9. A recent study by Martínez Portela et al. (2024) reported a significant association in this chromosome with sexual differentiation, along with strong linkage disequilibrium associated to a putative inversion. In our data, elevated differentiation on chromosome 9 occurs over multiple discrete regions rather than a single continuous block, suggesting local selection as the primary driver. The SRT‐box transcription factor 3 (sox3) has been proposed as the most compelling candidate gene for sexual determination, consistent with an XX/XY system. In our study, sox3 also appeared to be under selection when comparing samples from the North Sea and the Celtic Sea (File S1).

An alternative explanation to ecologically driven divergent selection is intrinsic genetic incompatibilities (Bierne et al. 2011). These are driven by negative interactions between genes that can occur both pre‐ and post‐zygotically. Under the coupling hypothesis, intrinsic genetic incompatibilities contribute to increased differentiation rather than local adaptation and act as endogenous barriers to gene flow, even in the absence of strong environmental differences. Similar findings have been reported for the deep‐water hake, where differentiation between males and females is driven by two major islands of differentiation in Chromosomes 1 and 2 (Gardiner et al. 2025).

### 4.3 Pleistocene Impact on the Demographic Dynamics of European Hake

We had data of sufficient resolution to infer for the first time the demographic history of European hake in the Northeastern Atlantic, governed by climatic and environmental changes over a time period from ca. 6 million years to 20,000 years ago. The most dramatic oscillations in population size in our study were observed in the late Pleistocene, likely coinciding with climate cycles. It is well‐established that the Pleistocene was an extremely dynamic era, with dramatic climatic fluctuations that strongly influenced the geographical distribution and population dynamics of both terrestrial and marine species (Hewitt 2004). The population decline observed in our study at ca. 300,000 years ago coincides with the Saale glaciation (130,000–300,000 years ago), where vast areas of northern Europe were covered by ice sheets and sea levels changed, possibly affecting coastlines and distribution of marine species (Hughes et al. 2020). The subsequent population increase coincides with the Eemian Interglacial (100,000–130,000 years ago), a period of climatic stability with temperatures up to 5°C warmer than today (NEEM 2013). During this period, higher sea levels lead to habitat increase for marine species, which could explain the population expansion observed in our study. The rapid increase in population sizes of European hake comes to a halt around 100,000 years ago, coinciding with the start of the Last Glacial Maximum, during which cold conditions prevailed and massive ice sheets covered large parts of the northern Hemisphere, including Scandinavia, the North Sea and mainland Europe (Svendsen et al. 2004). Many temperate species survived the last glaciation by retreating to ice‐free refugia in lower latitudes (Stewart et al. 2010). This could be the case of European hake, a species typically associated with warm environments. The species may have survived the last glaciation confined in localised refugia around the Iberian Peninsula, since the Celtic Sea and the North Sea were covered by ice. Towards the end of the Last Glacial Maximum, the ice sheets retreated from the Celtic Sea and the North Sea as the climate warmed, making these areas habitable once more for many temperate marine species that had been pushed to lower latitudes due to colder temperatures (Stroeven et al. 2016).

The PSMC suggests that the three hake populations reported here (North Sea, Celtic Sea, Portugal) were one single population until recently. The current population structure likely developed after the last glaciation, shaped by natural selection, local adaptation and possibly founder effects associated with post‐glacial colonisation. This is in accordance with the modest differences observed in our study when analysing all genomic markers but which become a much deeper genetic differentiation when considering only the subset of outlier markers. Because the populations are relatively young and have large effective population sizes, there has not been enough time to build a signature of differentiation by genetic drift. At the same time, selection for local adaptation appears strong enough to overcome the effects of contemporary gene flow. This suggests that strong divergent selection acting on environmentally relevant loci is the primary force initiating population divergence. While genetic drift has not yet produced genome‐wide signatures of differentiation, selection on traits associated with local environmental conditions has generated ‘islands of genetic differentiation’ across the genome. This short separation contrasts with the evolutionary history of anchovies, another species expanding in the North Sea, where PSMC analysis revealed a much older separation between populations, particularly the split of the South Portugal population, which occurred about 250,000 years ago during the late Pleistocene (Pujolar et al. 2025).

## 5 Conclusions

While the scope of the study was not to provide a comprehensive assessment of stock management for European hake in the northeastern Atlantic, given our limited sampling (i.e., we did not cover the full distribution of the species, particularly in the Bay of Biscay area), our findings indicate that the current ICES management of a single Northern stock may not fully reflect the actual population structure. The evidence for genetically distinct populations with signatures of local adaptation suggests a more complex scenario within the Northern stock, with at least partially distinct North Sea and Celtic Sea components. This substructure is relevant for current management but also for how the species may respond to future environmental changes. Among the predicted impacts of climate change are the geographic range shifts of many species, which could disrupt the established patterns of connectivity and gene flow among populations. These shifts may lead to changes in migratory routes and spawning sites, ultimately reshaping population structure. Therefore, fisheries management should consider the movement, mixing and exchange of individuals across populations and the importance of connectivity for preserving genomic diversity (Nielsen and Kenchington 2002; Reiss et al. 2009; Martinsohn et al. 2019). In fisheries management, it is crucial to recognise that many populations contribute to fisheries within a single stock and should be managed accordingly (Christiansen et al. 2022). Also, fisheries consisting of many populations tend to be more resilient to exploitation, as demonstrated in the case of sockeye salmon ( *Oncorhynchus nerka* ) in Alaska (Hilborn et al. 2003). This resilience is linked to the biocomplexity of fish stocks, consisting of distinct life‐history strategies and spatial distributions within a species, which acts as a buffer against both overfishing and environmental change. Ultimately, preserving the biocomplexity of the species as well as maintaining connectivity and gene flow are crucial for the adaptability of fish stocks in a rapidly evolving and dynamic environment.

## Author Contributions

**José Martin Pujolar:** data curation (lead), formal analysis (lead), investigation (lead), methodology (lead), visualization (lead), writing – original draft (lead), writing – review and editing (lead). **Courtney E. C. Gardiner:** formal analysis (supporting), methodology (supporting), writing – review and editing (supporting). **Sophie von der Heyden:** conceptualization (equal), funding acquisition (equal), writing – review and editing (supporting). **Joana I. Robalo:** conceptualization (equal), funding acquisition (equal), writing – review and editing (supporting). **Rita Castilho:** conceptualization (equal), funding acquisition (equal), writing – review and editing (supporting). **Regina L. Cunha:** conceptualization (equal), funding acquisition (equal), writing – review and editing (supporting). **Dorte Meldrup:** methodology (supporting), writing – review and editing (supporting). **Romina Henriques:** conceptualization (equal), formal analysis (supporting), funding acquisition (equal), investigation (supporting), methodology (supporting), project administration (equal), supervision (equal), writing – review and editing (supporting). **Einar E. Nielsen:** conceptualization (equal), formal analysis (supporting), funding acquisition (equal), investigation (supporting), methodology (supporting), project administration (equal), supervision (equal), writing – review and editing (supporting).

## Funding

This work was supported by the Department of Science and Innovation, South Africa, Project Biodiversity on the run: evolutionary and Innovationsfonden, BioDivClim Grant number 0156‐00018B (BioDiversa Ca Fundação para a Ciência e a Tecnologia, LA/P/0069/2020, LA/P/0101/2020, MARE/UIDB/MAR/04292, MARE/UIDP/04292, UIDB/04326/2020, UIDP/04326/2020).

## Ethics Statement

Fish samples were obtained through national fish monitoring surveys conducted by responsible authorities. No experimental procedures were conducted. Sampling complied with relevant ethical guidelines.

## Conflicts of Interest

The authors declare no conflicts of interest.

## Supporting information


**Table S1:** ece373085‐sup‐0001‐Tables.docx.

**File S1:** ece373085‐sup‐0002‐SupplementFile1.xlsx.

**Figure S1:** ece373085‐sup‐0003‐FigureS1.pdf.

## Data Availability

The data that support the findings of this study will be openly available on Genbank after publication, with all raw sequences submitted to the Sequence Read Archive SRA Submission SUB15499367. All code and scripts used for data analysis are available on Zenodo at https://doi.org/10.5281/zenodo.17912209.
