# Supplementary material for: Patterns of Population Genomic Variation and Evolutionary History of European Hake in the Northeastern Atlantic
Source: Ecol Evol. 2026 Feb 13;16(2):e73085. doi: 10.1002/ece3.73085 (PMC12905010; doi:10.1002/ece3.73085)
Supplement: Supplementary file 1 — Table S1: ece373085‐sup‐0001‐Tables.docx. [file ECE3-16-e73085-s002.docx]

**Supplementary Table 1.** Sampling details including sampling location, geographic coordinates, date, length (cm), weigth (g), sex and age.

| **Individual** | **Location** | **Date** | **L (cm)** | **W (g)** | **Sex** | **Age** | **Latitude** | **Longitude** | **Detailed location** |  |
| --- | --- | --- | --- | --- | --- | --- | --- | --- | --- | --- |
| H346 | Denmark | 27/08/2001 | 54 | 980 | M | 6 | 56.4 | 12.26 | Kattegat | |
| H381 | Denmark | 02/09/2021 | 37 | 364 | M | 2 | 57.21 | 11.66 | Kattegat | |
| H356 | Denmark | 28/08/2021 | 41 | 484 | F | 2 | 57.96 | 10.7 | Skagerrak | |
| H398 | Denmark | 04/10/2021 | 43 | 588 | - | 2 | 57.8 | 9.34 | Skagerrak | |
| H434 | Denmark | 12/10/2021 | 41 | 474 | - | 2 | 58.49 | 10.74 | Skagerrak | |
| H460 | Denmark | 11/10/2021 | 57 | 1370 | - | 4 | 58.63 | 9.38 | Skagerrak | |
| H018 | Denmark | 26/06/2021 | 64 | 1610 | F | 4 | 57.76 | 6.08 | Eastern North Sea, Denmark West | |
| H242 | Denmark | 23/07/2021 | 39.5 | 350 | M | 2 | 57.65 | 6.31 | Eastern North Sea, Denmark West | |
| H003 | Denmark | 20/08/2021 | 61 | 1558 | M | 4 | 57.46 | 8.61 | Eastern North Sea, Denmark West | |
| H004 | Denmark | 20/08/2021 | 60 | 1580 | M | 4 | 57.46 | 8.61 | Eastern North Sea, Denmark West | |
| H008 | Denmark | 20/08/2021 | 44 | 572 | F | 2 | 57.46 | 7.96 | Eastern North Sea, Denmark West | |
| H012 | Denmark | 22/08/2021 | 41 | 516 | M | 2 | 55.89 | 5.52 | Eastern North Sea, Denmark West | |
| H023 | Denmark | 29/06/2021 | 53 | 964 | - | 2 | 56.23 | 7.06 | Eastern North Sea, Denmark West | |
| H098 | N North Sea | 05/08/2021 | 24 | 96 | M | 1 | 56.63 | -1.63 | Northwestern North Sea, Scotland East | |
| H141 | N North Sea | 11/08/2021 | 45 | 640 | M | 2 | 59.08 | -1.39 | Northwestern North Sea, Scotland East | |
| H249 | N North Sea | 26/07/2021 | 57 | 1250 | M | 2 | 57.74 | 1.27 | Central Northern North Sea | |
| H154 | N North Sea | 13/08/2021 | 67 | 1770 | F | 4 | 60.55 | 2.11 | Central Northern North Sea | |
| H268 | N North Sea | 30/07/2021 | 57 | 1070 | F | 2 | 59.31 | 2.3 | Central Northern North Sea | |
| H299 | N North Sea | 04/08/2021 | 74 | 2510 | F | 4 | 60.27 | 2.61 | Central Northern North Sea | |
| H313 | N North Sea | 06/08/2021 | 37 | 322 | M | 2 | 60.69 | 0.85 | Central Northern North Sea | |
| H343 | N North Sea | 09/08/2021 | 39.5 | 420 | F | 2 | 61.55 | 0.61 | Central Northern North Sea | |
| H191 | Ireland | 21/11/2021 | 43 | 497 | M | 2 | 58.07 | -9.02 | Ireland North | |
| H199 | Ireland | 23/11/2021 | 42 | 498 | M | 2 | 56.34 | -9.07 | Ireland North | |
| H216 | Ireland | 02/12/2021 | 42 | 528 | F | 2 | 56.38 | -7.66 | Ireland North | |
| H020 | Ireland | 02/11/2021 | 50 | 937 | F | 2 | 56.36 | -8.34 | Ireland North | |
| H465 | Ireland | 2021 | 52 | - | - | 2 | 51.46 | -14.72 | Porcupine Bank, Ireland West | |
| H472 | Ireland | 2021 | 53 | - | - | 2 | 51.69 | -14.66 | Porcupine Bank, Ireland West | |
| H478 | Ireland | 2021 | 69 | - | - | 4 | 52.2 | -14.67 | Porcupine Bank, Ireland West | |
| H482 | Ireland | 2021 | 44 | - | - | 2 | 52.38 | -14.83 | Porcupine Bank, Ireland West | |
| H507 | Ireland | 2021 | 66 | - | - | 4 | 53.79 | -11.95 | Porcupine Bank, Ireland West | |
| H517 | Ireland | 2021 | 60 | - | - | 4 | 53.8 | -11.59 | Porcupine Bank, Ireland West | |
| H180 | Ireland | 28/11/2021 | 63 | 1691 | F | 4 | 50.65 | -7,62 | Celtic Sea, Ireland South | |
| H201 | Ireland | 30/11/2021 | 70 | 2452 | F | 4 | 51.25 | -8.05 | Celtic Sea, Ireland South | |
| H253 | Ireland | 10/12/2021 | 40 | 441 | M | 3 | 51.19 | -6.64 | Celtic Sea, Ireland South | |
| H026 | Portugal | 09/12/2020 | 32.5 | 279 | F | 2 | 38.56 | -9.41 | Lisbon Coast, Portugal West | |
| H028 | Portugal | 09/12/2020 | 33.6 | 228 | F | 2 | 38.56 | -9.41 | Lisbon Coast, Portugal West | |
| H038 | Portugal | 09/12/2020 | 36 | 319 | M | 2 | 38.56 | -9.41 | Lisbon Coast, Portugal West | |
| H057 | Portugal | 21/06/2021 | 34.4 | 271 | F | 2 | 36.84 | -8.25 | Algarve, Portugal South | |
| H063 | Portugal | 24/06/2021 | 35 | 325 | F | 2 | 36.77 | -7.83 | Faro, Portugal South | |
| H076 | Portugal | 27/06/2021 | 47 | 942 | F | 2 | 36.97 | -7.51 | Gulf of Cadis, Portugal South | |

**Supplementary Table 2.** Summary of genomic variabily including nucleotide diversity (π) and individual heterozygosity (H_O_) detailed for each sampling location and for the North Sea, Celtic Sea and Portugal clusters. SD= Standard deviation.

| **Location** | **Π** | **SD** | **H_O_** | **SD** |
| --- | --- | --- | --- | --- |
| Denmark | 0.2550 | 0.1845 | 0.1616 | 0.0037 |
| N North Sea | 0.2591 | 0.2055 | 0.1690 | 0.0110 |
| Ireland | 0.2581 | 0.2071 | 0.1644 | 0.0073 |
| Portugal | 0.2631 | 0.1876 | 0.1767 | 0.0087 |
|  |  |  |  |  |
| North Sea cluster | 0.2540 | 0.1614 | 0.1618 | 0.0043 |
| Celtic Sea cluster | 0.2560 | 0.1556 | 0.1654 | 0.0100 |
| Portugal cluster | 0.2631 | 0.1876 | 0.1767 | 0.0087 |

**Supplementary Table 3.** Cross-validation (CV) error estimates from admixture analyses for each tested number of clusters (K= 1-6) across three datasets (all SNPs, neutral SNPs, and outlier SNPs), calculated using 10-fold cross-validation (CV = 10).

| **K** | **Outlier SNPs** | **Neutral SNPs** | **All SNPs** |
| --- | --- | --- | --- |
| 1 | 0.52275 | 0.45107 | 0.45239 |
| 2 | 0.51531 | 0.52919 | 0.53164 |
| 3 | 0.51466 | 0.61800 | 0.60146 |
| 4 | 0.58582 | 0.69127 | 0.68306 |
| 5 | 0.68722 | 0.75985 | 0.75555 |
| 6 | 0.76127 | 0.83082 | 0.83796 |

**Supplementary Table 4.** Evidence for selection in genes involved in osmoregulation, including chromosome location, gene start and end position and genetic differentiation (F_ST_) value.

| **Sodium/Potassium-transporting ATPase Subunit alpha (ATP1A1a)** | | |  |
| --- | --- | --- | --- |
| Chromosome | Start | End | F_ST_ |
| 6 | 27059142 | 27105072 | 0.0096 |
| 14 | 9629565 | 9653823 | 0.02 |
| 19 | 3636172 | 3678585 | 0.0042 |
| **Sodium/Potassium-transporting ATPase Subunit beta (ATP1A1b)** | | |  |
| Chromosome | Start | End | F_ST_ |
| 1 | 40674290 | 40397131 | 0.0102 |
| 2 | 4507389 | 4517193 | 0 |
| 2 | 13515084 | 15530900 | 0.0117 |
| 3 | 6228050 | 6268089 | 0.0033 |
| 3 | 38900022 | 38905016 | 0 |
| 6 | 11964831 | 11973981 | 0 |
| 8 | 15409768 | 15413136 | 0.0503 |
| 9 | 15544838 | 15550843 | 0.1523 |
| 11 | 5877066 | 5886730 | 0.0638 |
| 17 | 23449894 | 23462061 | 0.0017 |
| 19 | 11304302 | 11313122 | 0.0569 |
| 21 | 15145524 | 15208099 | 0.0045 |
| **Na(+)/H(+) exchange regulatory co-factor (NHE)** | | |  |
| Chromosome | Start | End | F_ST_ |
| 2 | 26309246 | 26423902 | 0.0328 |
| 2 | 34582946 | 34611991 | 0.0218 |
| 3 | 22844343 | 22850154 | 0.0216 |
| 4 | 12295647 | 12301335 | 0 |
| 5 | 4569047 | 4582711 | 0.0016 |
| 13 | 21672808 | 21689322 | 0.0082 |
| 17 | 22860853 | 22869141 | 0 |
| **V-type proton ATPase** | |  |  |
| Chromosome | Start | End | F_ST_ |
| 1 | 11329189 | 11330406 | 0.0041 |
| 2 | 34490180 | 34493727 | 0 |
| 2 | 43666011 | 43685930 | 0.0508 |
| 4 | 26582965 | 26584937 | 0 |
| 6 | 21325148 | 21345241 | 0 |
| 8 | 28117815 | 28173683 | 0.0041 |
| 9 | 8593516 | 8594094 | 0.22 |
| 9 | 21527613 | 21547084 | 0.1037 |
| 10 | 5993244 | 6004872 | 0 |
| 12 | 10876490 | 10884040 | 0.014 |
| 13 | 6143487 | 6154164 | 0.007 |
| 13 | 22116219 | 22140321 | 0 |
| 14 | 27424890 | 27433087 | 0.0044 |
| 16 | 254308 | 259727 | 0 |
| 16 | 15460672 | 15471576 | 0.0032 |
| 19 | 10369263 | 10376208 | 0.008 |
| 21 | 7498219 | 7504183 | 0.0215 |
| **Carbonic Anhydrase** | |  |  |
| Chromosome | Start | End | F_ST_ |
| 2 | 32083351 | 32162297 | 0.0135 |
| 3 | 19462747 | 19473144 | 0.0005 |
| 5 | 21741830 | 21744110 | 0 |
| 6 | 77933806 | 7946777 | 0.0191 |
| 6 | 29121238 | 29142380 | 0.0555 |
| 8 | 13207219 | 13213937 | 0 |
| 13 | 6413745 | 6420016 | 0 |
| 13 | 6450918 | 6480335 | 0.056 |
| 13 | 21479603 | 21612049 | 0.0029 |
| 14 | 5756483 | 5767205 | 0.0019 |
| 15 | 6831253 | 6847192 | 0 |
| 15 | 16401158 | 16413015 | 0.0198 |
| 16 | 15841538 | 15845633 | 0 |
| 17 | 5170785 | 5177217 | 0.0003 |
| 17 | 13834422 | 13843614 | 0.0038 |
| 18 | 25460919 | 25469190 | 0 |
| **Acid-sensing (proton-gated) ion channel** | | |  |
| Chromosome | Start | End | F_ST_ |
| 2 | 37266639 | 37267533 | 0 |
| 3 | 8895784 | 8934485 | 0.0179 |
| 6 | 17561652 | 17636744 | 0.025 |

**Supplementary File 1.** Candidate list of all annotated genes putatively under selection including gene name, gene description, gene location, gene start and end position and summaries from Entrez and UniProtKB/Swiss-Prot.
