# Supplementary material for: Patterns of Population Genomic Variation and Evolutionary History of European Hake in the Northeastern Atlantic
Source: Ecol Evol. 2026 Feb 13;16(2):e73085. doi: 10.1002/ece3.73085 (PMC12905010; doi:10.1002/ece3.73085)
Supplement: Supplementary file 3 — Figure S1: ece373085‐sup‐0003‐FigureS1.pdf. [file ECE3-16-e73085-s001.pdf]

Supplementary Figure 1. Visualisation of population structure using a Principal Component Analysis (PCA) of all 40 individuals using the subset of Neutral SNPs. Each point represents an individual. Dashed circles are manually drawn to indicate the groups identified, for visual guidance only.

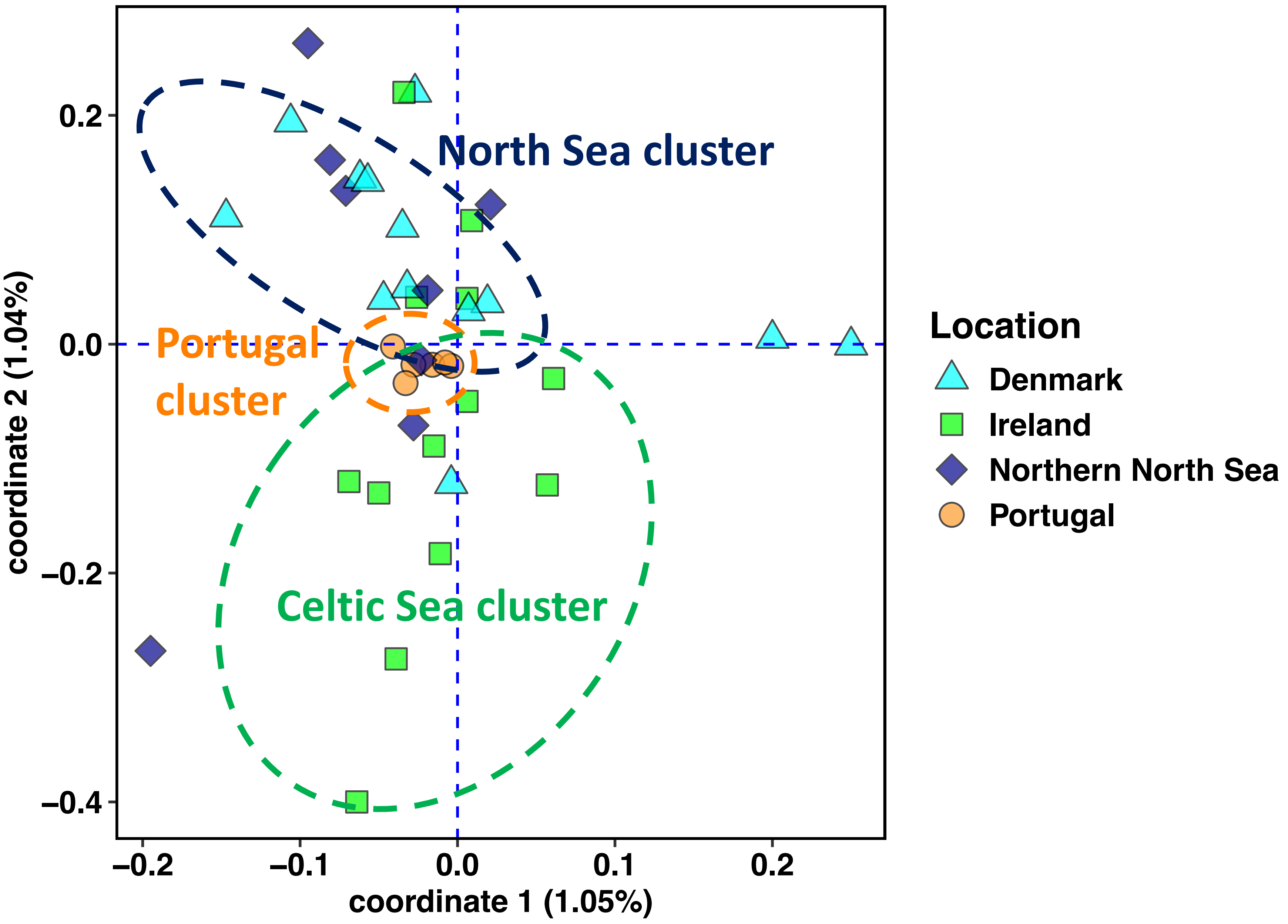
North Sea cluster
Portugal
cluster
Celtic Sea cluster
0.2
0.0
−0.2
−0.4
coordinate 2 (1.04%)
−0.2
−0.1
0.0
0.1
0.2
coordinate 1 (1.05%)
Location
Denmark
Ireland
Northern North Sea
Portugal
